# Icing in the Cake: Water in Nanoscopic Confinement by Cellulose

**DOI:** 10.1021/acs.jpcb.5c06900

**Published:** 2025-11-19

**Authors:** Alíz Lelik, Lars Berglund, István Furó, Jakob Wohlert

**Affiliations:** † Department of Fiber and Polymer Technology, 7655 KTH Royal Institute of Technology, SE-10044 Stockholm, Sweden; ‡ Department of Chemistry, KTH Royal Institute of Technology, SE-10044 Stockholm, Sweden; § Wallenberg Wood Science Center, KTH Royal Institute of Technology, SE-10044 Stockholm, Sweden

## Abstract

Water has profound effects in both cellulosic materials and the plant cell wall. In particular, water was recently shown to reside at the fibril–fibril interfaces inside cellulose fibril aggregates where it attains a structural role. We use molecular dynamics simulations to investigate the properties of water confined by cellulose surfaces at a specific and conceptually well-defined distance *L*. We study different crystalline faces of cellulose interacting with the water molecules and vary the confinement so that the water region changes from submonomolecular to essentially bulk. We find that confinement hinders molecular motions. In particular, the translational self-diffusion coefficient *D* exhibits a dramatic divergence and slows by up to three orders of magnitude from its bulk value for a defective monolayer of water. In the same regime, water also attains a strong preferential orientation with regards to the confining surfaces. The mass density of the water layer evolves with *L* in a nonmonotonic and intriguing manner. As pore size decreases, at roughly monolayer separation, the density first increases from its bulk value so that it approaches the densities of high-pressure forms of ice. When water becomes sparser than monomolecular, its mass density sharply drops as it should for a defective layer. In this defective layer, the reorientation is not only slow, but completely anisotropic. These observations on the atomistic scale highlight the unique ways cellulose and water, two very abundant materials interact with each other.

## Graphical abstract

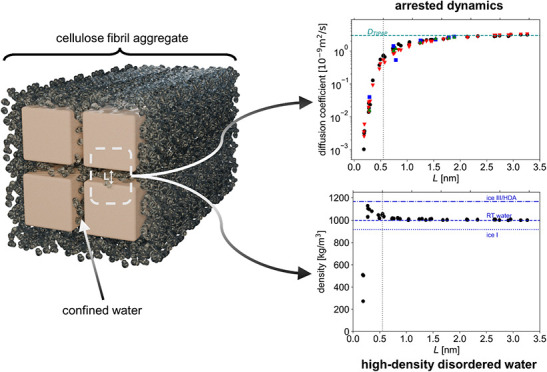

## Introduction

Adsorbed water is often considered a problem in polymeric materials science, because of plasticization and softening of the polymer. Especially so, it is a consideration for materials based on cellulosic plant fibers. Due to their chemical composition, in particular the large abundance of polar hydroxy groups, cellulose-containing fibers are hygroscopic. They adsorb moisture when exposed to humid or wet environments, which leads to swelling and softening of the fibers, compromising both dimensional stability and mechanical properties of the material.^1–4^ High moisture content also accelerates decay by chemical means (hydrolysis) or by microorganisms.^5,6^

On the other hand–wood is a hydrated natural fiber composite. In its native state the load-bearing structure (the wood cell wall) is saturated with moisture, containing more than 20% water by weight.^7^ Still trees can grow hundred meters tall and stand for a thousand years. Unlike in synthetic materials, mechanical properties are not as compromised upon hydration, in some cases even a strengthening effect is observed.^8,9^ Thus, cellulose-water interactions can be both an asset or a detriment, depending on the perspective one takes, as argued by several recent reviews.^7,10,11^ A related experimental observation is that complete drying of cellulosic substrates is very challenging–as an example, near complete removal of residual water can take up to a week under continuous flow of dry nitrogen.^12,13^

Hindered dynamics of water is shown by nuclear magnetic resonance (NMR) spectroscopy^14,15^ and quasi-elastic neutron scattering in both wood and man-made cellulosic products.^16–18^ One particularly interesting observation comes from ^2^H NMR on cotton linters.^13^ These experiments all show a distinct population of water molecules in very slow exchange with the rest of the water in cellulose, exhibiting dynamics that is both anisotropic and orders of magnitude slower than liquid water at the same temperature. This population remains weakly affected by drying and is thus identified as water molecules confined in the nanoscale cavities between cellulose microfibrils, which were previously considered inaccessible. This assignment is supported by MD simulations,^19,20^ which also show that such interfacial water molecules are thermodynamically stable, and not merely kinetically trapped;^20^ and that by the presence of this interfacial water, the free energy of the fibril aggregate is decreased. It is also shown that interfacial water increases the work required to separate two cellulose nanocrystals,^21^ indicating that confined water contributes to the strength and stability of a fibril aggregate and can be considered a structural element of the cellulose material.^22–24^ This is akin to the role of buried water in some proteins which are essential for the native structure and thus their biochemical function.^25,26^

Atomistic simulations show that confinement has significant effects on both water structure and dynamics in a wide range of systems. Many studies focus on proteins, due to their biological relevance;^27^ or inorganic substrates, such as hydrophilic silica^28–31^ and graphene,^32,33^ with specific attention toward how properties depend on the distance of the confining surfaces and their chemistry,^34,35^ but relevant works involving cellulose are rare. One notable exception is O’Neill et al.^16^ who show that water confined by cellulose fibrils exhibits much slower translational diffusion than liquid water at the same temperature. In addition, the distribution of diffusion coefficients is broad, spanning at least 1 order of magnitude.

The slit-pore geometry is a useful and commonly utilized framework to study confinement. One particular study shows that the average translational self-diffusion of water confined between two infinitely large silica platelets decreased by a factor of 3 compared to liquid state at a platelet separation of 0.5 nm.^36^ The experimental observations on the pore-size dependence of dynamics in mesoporous silica align well with the slit-pore simulations.^37^ Although this effect is significant, it becomes less so in light of the two-order-of-magnitude decrease observed in experiments for water in cellulose pores. This implies that the chemical structure of the surface may play a significant role, in addition to effects from the confinement itself.

Given the fact that water affects cellulose materials in complex and somewhat unpredictable ways, paired with the indications that water confined in cellulose behaves vastly different from liquid water, it is of interest to study these phenomena in greater detail. To that end, the present study employs MD simulations to model physical properties of water molecules confined by infinitely large cellulose surfaces forming a system with a slit-pore geometry. This system, combined with a rigorous definition of *L*, a measure of confinement, makes it possible to isolate how water dynamics depend on the separation between the confining planes. The investigation is inspired by NMR results, where mechanisms for water dynamics can be characterized in great detail. The hypothesis is that both dynamics and structure are affected. This hypothesis is systematically investigated by varying both the confinement and the nature of the confining cellulose surfaces. In the broader perspective, possible effects on macroscale material properties are discussed (Figure 1).

**Figure 1:**
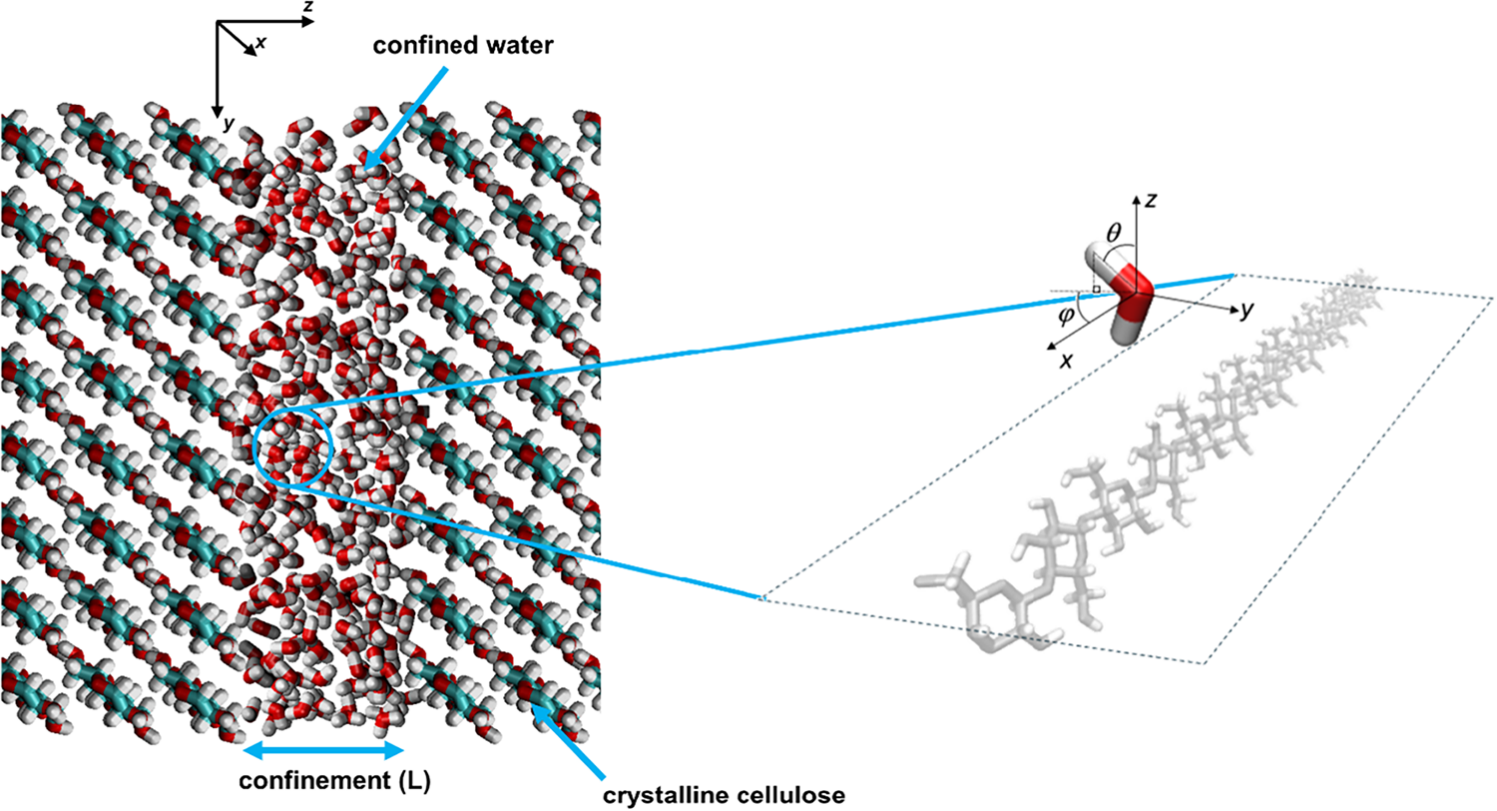
Slit-pore geometry comprised of (110) cellulose confining surfaces. The schematic on the right highlights the principal directions and the OH bond angles with regards to the cellulose chains in the confining surfaces.

## Methods

Molecular dynamics simulations were performed using GROMACS 2023.2.^38^ A cutoff of 1.0 nm was used to treat nonbonded interactions and the PME method was used to include long-range electrostatics.^39,40^ The simulations were 10 ns long after equilibration, with a basic time step of 2 fs, saving coordinates every 2 ps. A temperature of 300 K and pressure of 1 bar was maintained using a stochastic velocity rescaling thermostat and a semi-isotropic cell-rescaling barostat, including a stochastic term.^41,42^ This means that the pressure was controlled separately in the *xy*- and *z* directions. All bonds involving hydrogen atoms were constrained using the P-LINCS algorithm.^43^

The cellulose models were generated using the cellulose-builder tool,^44^ using the GLYCAM06 carbohydrate force field.^45^ Each cellulose structure was composed of chains with a length of 4 cellobiose units, containing 32 or 36 of these chains arranged to form Iβ-cellulose with the (110), (1–10) and (100) crystalline faces exposed toward the slit. This is the crystalline form most commonly found in higher plants; most importantly, in the wood cell wall.^46,47^ These cellulose slabs were placed in simulation boxes with periodic boundary conditions enabled in all directions, creating an infinite crystalline cellulose surface. Water was added in the slit pore, after which the system was equilibrated for 0.25 and 0.5 ns at constant volume and constant pressure, respectively. During the second step the distance between the cellulose surfaces was not fixed. Comparisons to bulk properties were made based on a simulation of a 4 nm × 4 nm × 4 nm large box of TIP4P water at the same thermodynamic conditions as the cellulose-water simulations.

In each simulation run, the cellulose structures were arranged to create a system where varying amounts of water is confined between two infinite cellulose surfaces. Both in plant cell walls and in technically relevant celluloses (MFC and nanocellulose) association of hydrophilic cellulose surfaces is prevalent,^48^ but in order to assess the effect of the hydrophobicity of the surface on the confinement effects, a set of different arrangements were chosen: identical, parallel (110) cellulose surfaces, identical (110) crystalline faces in antiparallel arrangement, (110) and (1–10) (hydrophilic) crystalline faces in parallel, or identical (100) (hydrophobic) faces, also in parallel. Note that the most irregular system is the one with the (110) face opposite to the (1–10), where an 8-chains-wide face meets a 9-chains-wide face. For each of these arrangements, 14 different confinements in the range of *L* = 0.2–3.5 nm between the crystalline surfaces were simulated. The spacing (if any) between microfibrils in the plant wall is not well-known; different experimental methods of this estimation yield a wide range of results;^49,50^ but nearly all of them fall under 1 nm, with several estimates around 0.5 nm. Confinement however does have an effect in pores larger than 1 nm too,^51^ thus the choice of a wider range.

## Results and Discussion

In this study, water dynamics is characterized by translational self-diffusion and local reorientation dynamics, and its structural characteristics via the preferential orientation and local density of water molecules across the confined water layer. The extent of confinement was used as a basis of comparison between systems, as this was previously shown to influence both water dynamics and structure in graphene slit-pores.^52^

### Defining the Geometric Confinement

The seemingly simple task of defining the geometric width *L* of the central water slab turns out to be rather complicated due to the ambiguities that arise from molecular surface roughness and the use of soft potentials. Consequently, there are many suggestions how to define *L*, as is detailed in the Supporting Information. There are two basic options, either (i) use cellulosic atoms in the two opposing slabs to set limits or (ii) to use the properties of water molecules to the same effect. Option (ii), based on the properties of the water molecules attempts to overcome the problems arising in option (i) from that not necessarily all volume not available for cellulose is available for water, but generally either option suffers from being somewhat arbitrary. Below, we propose an alternative: a geometric parameter obtained from allowing water molecules themselves to explore the available volume. To that end, we evaluate the time evolution of the mean square displacement (MSD) of the confined water molecules. Governed by self-diffusion with coefficient *D*, this can proceed in two directions parallel (*x* and *y*) and one direction perpendicular (*z*) to the confining surfaces (Figure 1), which are treated separately. For parallel diffusion, the MSD in the time limit beyond the collision time scale is given by the Einstein relation,

1 $$\left\langle x^{2}\right\rangle =2D_{∥,x}t$$

2 $$\left\langle y^{2}\right\rangle =2D_{∥,y}t$$

where ⟨*x* ^2^⟩ and ⟨*y* ^2^⟩ are the MSDs in the two parallel directions with the corresponding parallel self-diffusion coefficients. Indeed, those MSDs are linearly proportional to the diffusion time as shown in Figure 2, as the diffusion is unrestricted. There was no significant difference between *D*_∥,*x*_ and *D*_∥,*y*_ (see Figure S1 for a comparison), and in the following they will simply be referred to as their composite value *D*_∥_.

**Figure 2:**
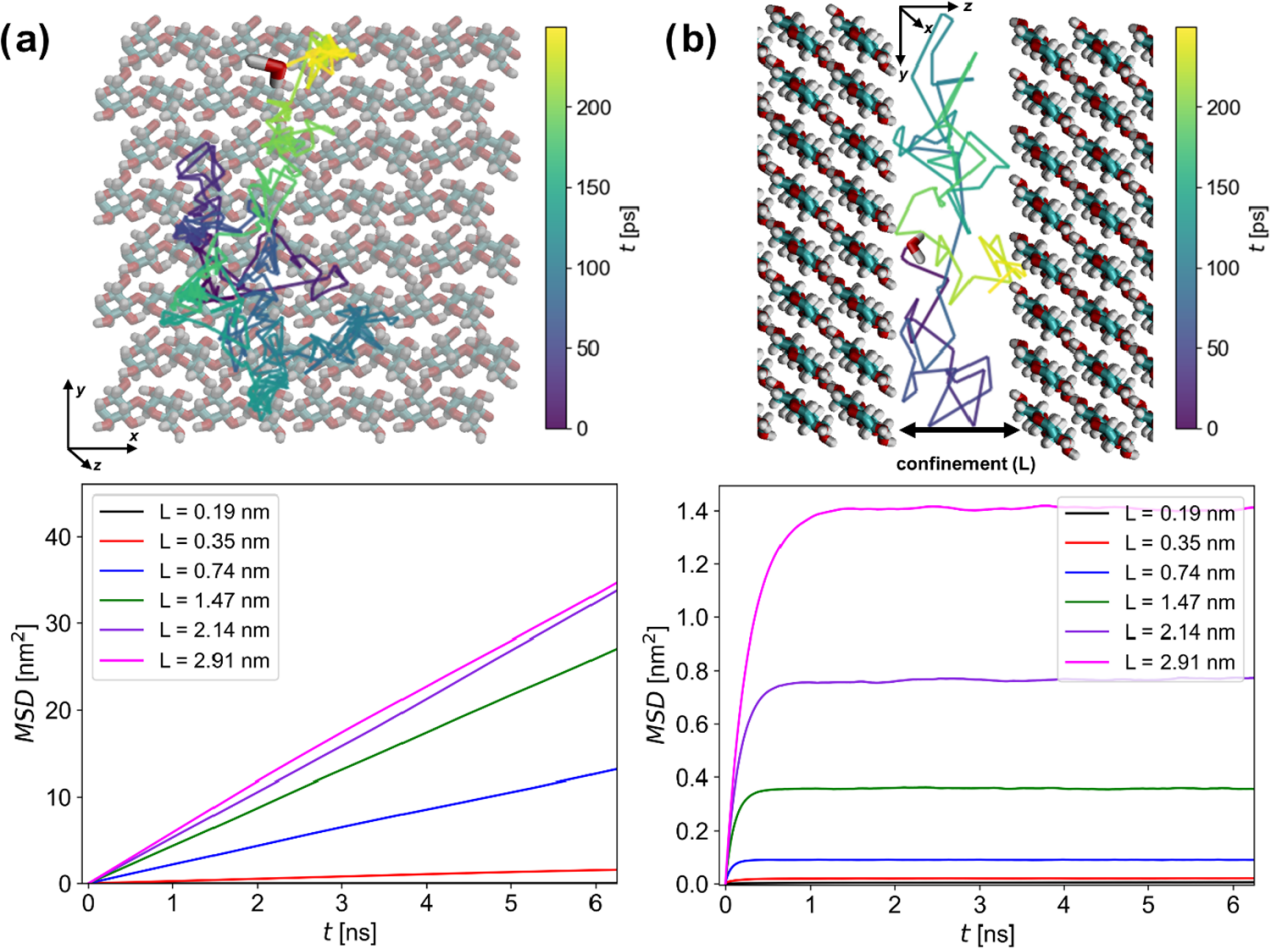
Mean square displacements of the water molecules parallel (a) and perpendicular (b) to the cellulose surface as a function of time, at different confinement, with representative examples of the corresponding trajectories in relation with the cellulose surface. The definition of *L* in these graphs is based on eq 3 below.

The MSD in the perpendicular direction is nonlinear, and it quickly levels off to a constant value, since it is restricted by the confining surfaces (Figure 2b). As discussed, *L* cannot simply be defined as the distance between surfaces, therefore we seek a macroscopic parameter characterizing this restriction. The known solution to the Fick equation of diffusion for the case of boundary conditions representing two, impenetrable, reflective walls separated by a distance *L* yields^53^

3 $$\left\langle z^{2}\right\rangle =\frac{L^{2}}{6}\left(1-\frac{48}{\pi^{2}}\sum_{n=1}^{\infty }\frac{1-{\left(-1\right)}^{n}}{n^{4}}\exp\left(\frac{-D_{⊥}n^{2}\pi^{2}t}{L^{2}}\right)\right)$$

where *D*_⊥_ is the self-diffusion coefficient in the perpendicular direction. From this expression it is clear that the plateau in the MSD at large *t* can be identified as *L* ^2^/6, regardless of the value of *D*_⊥._ Therefore, *L* and *D*_⊥_. were treated as independent parameters and are thus fitted to the simulation data in Figure 2b simultaneously. The functional variation is such that the covariance of these two parameters is negligible as is shown by a Monte Carlo correlation analysis (see SI). Thus, eq 3 does not only present a computationally accurate way to calculate *D*_⊥_, it also presents a straightforward method to establish the separation *L* as a quantitative and singular geometric measure of the confinement in the system.

### Confinement Slows Down Water Diffusion

Self-diffusion coefficients are plotted in Figure 3, as a function of confinement by different cellulose faces. Interestingly, the hydrophilic and hydrophobic faces of cellulose seem to affect the mobility of water in the same way. With decreasing distance between the confining planes, *D*_∥_ increasingly deviate from the bulk liquid value of the TIP4P model^54^ of *D* = 2.95·10^–9^ m^2^/s. This trend is consistent with the experimental trends of water self-diffusion coefficients in model cell walls at different levels of hydration^55^ as well as with simulation trends observed in hydrophobic slit-pore confinement,^52^ which show suppressed water mobility with increasing confinement. The obtained strong variation is of particular interest in light of the experimental data for cellulose interfibril separation in the nanometer to subnanometer range in cellulose fibril aggregates found in plant cells and in cellulosic materials.^49,50,56,57^

**Figure 3:**
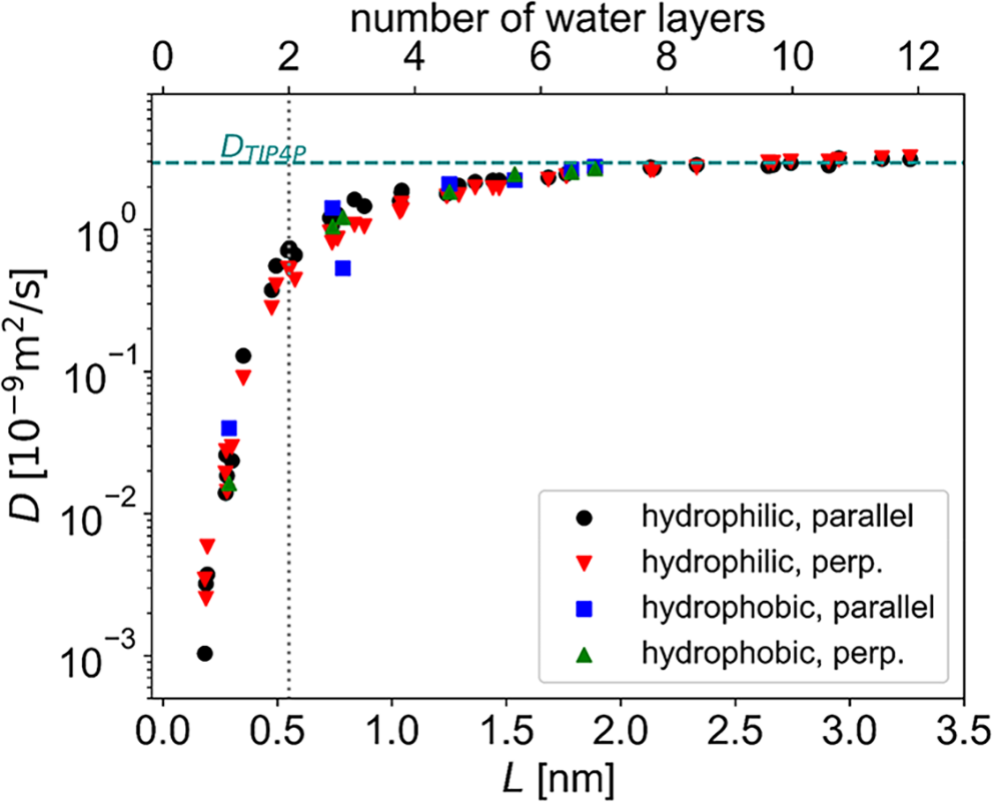
Calculated self-diffusion coefficients as a function of the confinement, parallel and perpendicular to the confining surfaces. The transition from two distinct layers of water to a more bulk-like state is marked with a dotted line.

Estimates from ^2^H MAS NMR measurements predict a residence time in the order of one microsecond for water in interfibrillar spaces in hydrated cellulose systems,^13^ which corresponds to a self-diffusion coefficient in the order of 10^–13^ m^2^/s. Pulse-field gradient NMR measurements yield a higher estimate, 10^–10^ to 10^–11^ m^2^/s at low hydration.^14^ We find that translational molecular motions slow down 3 orders of magnitude compared to the bulk liquid at the highest confinements (smallest *L*, corresponding to a defective monolayer of water). This is reasonably close to the experimental indications, especially considering that we compare simulations of model systems with diverse experimental values recorded at different water contents, in systems with wide distributions of pore sizes and shapes, where faster motions in larger pores may dominate the average measured. These findings highlight how strong the effect of pore size is on the mobility of water in fiber networks–similar to that observed in inorganic porous materials^58^—and could potentially be used to experimentally identify different dynamics in differently sized pores.

Studies concerning diffusion of water in confined spaces often report subdiffusion, characterized by an S-shaped curve for the three-dimensional MSD,^30,34,59^ The results here show that in the slit-pore geometry, the lateral and perpendicular directions can be treated separately, and in the unrestricted parallel directions, the MSD appears perfectly linear. We find no difference in the mobility of the water molecules in the two directions parallel to the cellulose, in spite of the anisotropy of the surfaces. Despite confinement, the difference between the parallel and perpendicular self-diffusion coefficients in Figure 3 is small (comparable with the standard deviations of the data set) compared to the effect of confinement, which is orders of magnitude higher. This is the typical behavior of water in hydrophilic confinement, as opposed to that in hydrophobic confinement in materials other than cellulose, where diffusion perpendicular to the confining walls was shown to be faster than parallel diffusion.^60^ In addition, we obtained similar behavior for hydrophilic and hydrophobic faces of cellulose.

### Confinement Slows Down the Reorientational Dynamics of Water

Alongside the translational diffusion, the rotational mobility of the water molecules can be characterized by the rotational autocorrelation functions (RACFs) of the water O–H bonds

4 $$C\left(t\right)={\left\langle P_{2}\left(\cos\left[\theta \left(t_{0},t_{0}+t\right)\right]\right)\right\rangle }_{t_{0}}$$

where *P*_2_ is the second-order Legendre polynomial and θ­(*t*_0_, *t*_0_ + *t*) is the angle between the O–H bond vector at time *t*_0_ and a time *t* later. The subscript means that the average is calculated over all possible time origins. The RACFs of the water molecules at different, selected confinements are presented in Figure 4.

**Figure 4:**
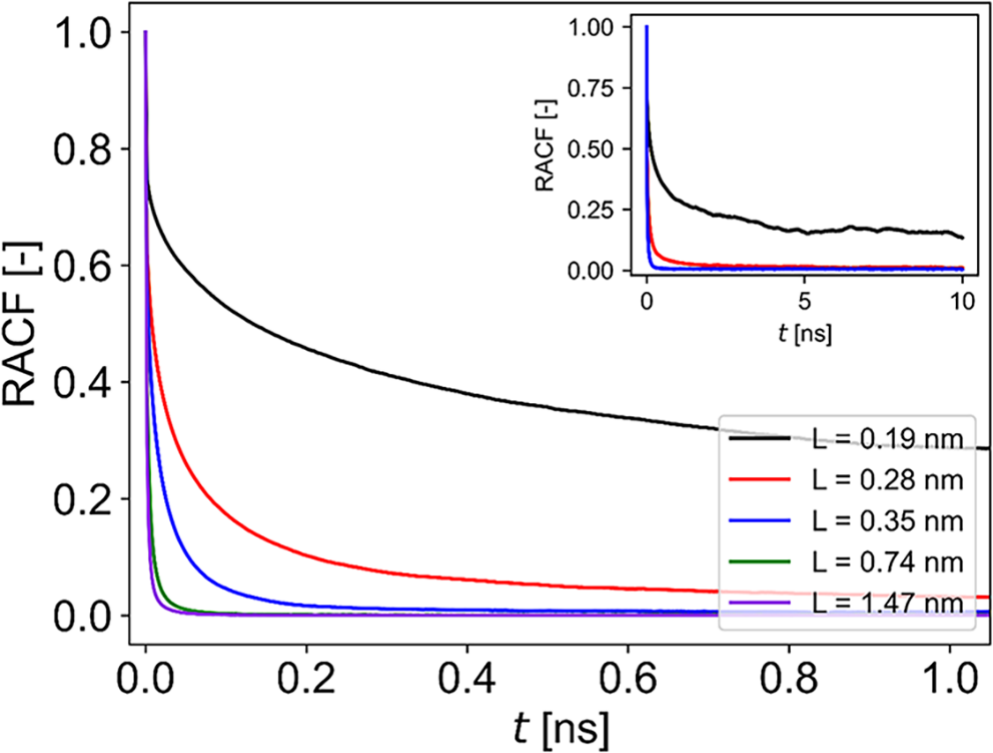
Water mobility expressed as decay of the rotational autocorrelation functions of water at different confinements. The inset shows the behavior of the most confined systems over longer time scales.

The calculated RACFs for water mobility exhibit complex nonexponential decays, similar to what was observed before in simulations of water molecules confined in proteins.^27,61^ Thus, in order to meaningfully compare the curves obtained at different *L*, the data were fitted with a stretched exponential function

5 $$C\left(t\right)=S^{2}+\left(1-S^{2}\right)e^{-{\left(t/\tau \right)}^{\beta }}$$

in which, the magnitude of the reorientational correlation time τ describes the correlation time scale; lower τ values correspond to higher mobility. The exponent β is a measure of complexity, while the order parameter *S* corresponds to the long-time plateau of the RACF (Figure 4 inset) and gives a measure of the remaining anisotropy of water molecules in the system, which is not averaged out by reorientational motion. In systems with no anisotropy, such as bulk liquid water, *S* becomes negligibly small. Oleinikova et al.^27^ modeled reorientational dynamics as an average over two populations: one hindered with a stretched exponential decay and one freely reorienting with a monoexponential decay. With this model they obtained good fits at all levels of hydration. However, distinguishing between such populations is problematic because of the fast exchange between them and as discussed below, the decay is not necessarily purely monoexponential even for liquid water.

Figure 5 shows τ, β, and *S* as a function of confinement, with the corresponding reorientational correlation time τ from a bulk simulation included. All parameters approach their bulk values with decreasing confinement, with τ reaching its bulk value at around *L* = 1.5 nm. This slowing down of the rotational motions in the simulations is consistent with earlier findings based on ^2^H NMR measurements on hydrated cellulose,^13,20^ where a connection between hydration level and dynamics was made in amorphous cellulose systems.^62^ The difference between the rotational correlation times with hydrophilic and hydrophobic faces appears to be small, just as we have observed for translational diffusion.^63^ This is not anticipated and, indeed, curious, since the difference in dynamics between hydrophilic and hydrophobic confinements is notable in inorganic materials. This clearly calls for more detailed future investigations.

**Figure 5:**
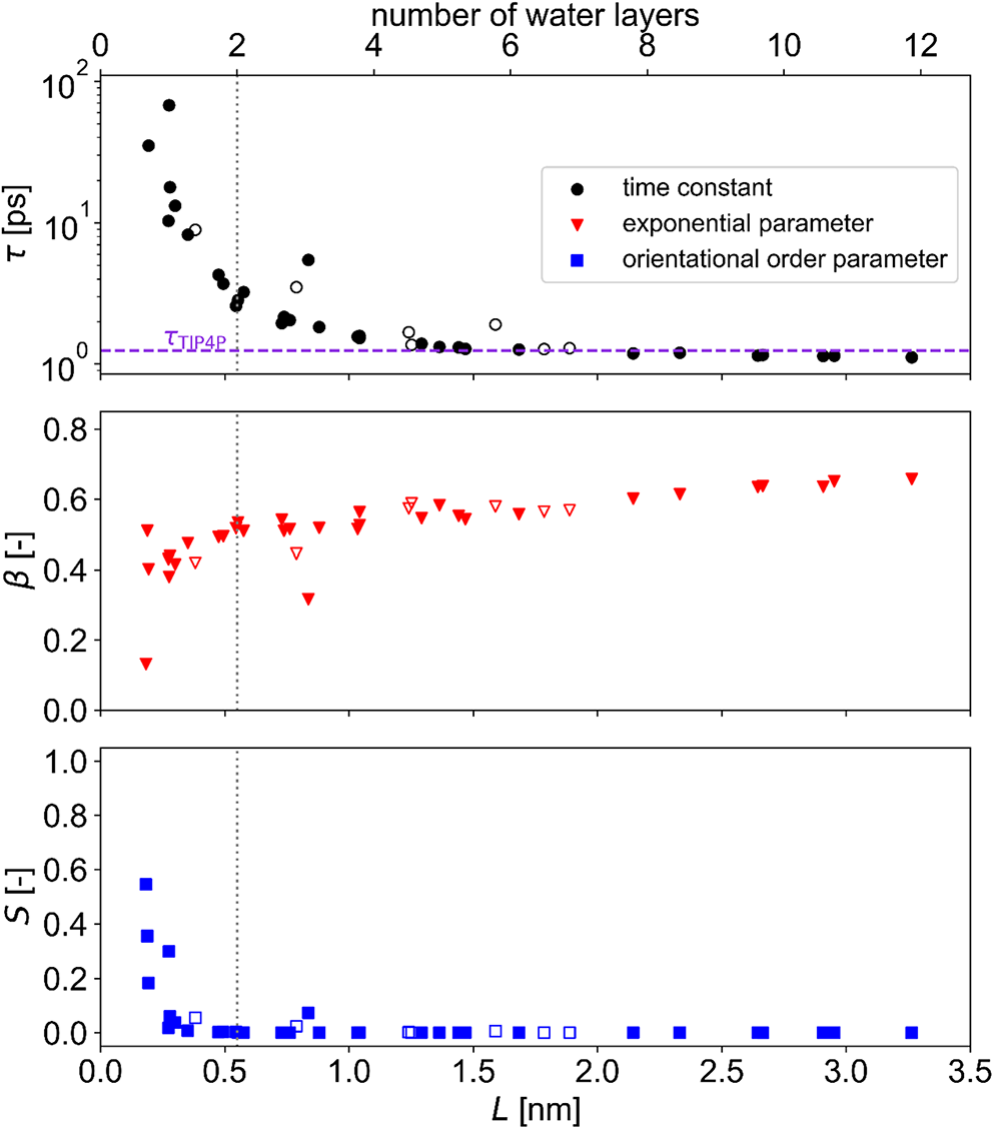
Fitting parameters of the RACFs (see eq 5) as a function of the extent of confinement. Standard deviations are comparable to the size of the markers; empty markers represent confinement by hydrophobic cellulose.

The parameter β increases with increasing *L*. This indicates that as the mobility increases, the reorientational dynamics becomes simpler, with confinement becoming locally irrelevant with increasing amount of space available. However, the β = 1 limit (that is, pure monoexponential decay), is not reached. Peculiar as it may seem, this is primarily due to the fact that the reorientation of water molecules in bulk liquid water is not strictly monoexponential either, but rather follows a complex molecular jump mechanism steered by the hydrogen-bond network.^64^

For highly confined water, the value of the order parameter *S* shows a sharp increase with less available space for the water molecules, indicating that there is a weak, but persisting correlation even at long-term between the original and the current orientation of the OH bonds. This long-time order is the result of the presence of significant anisotropic local potentials in form of the two confining cellulose layers, not only a consequence of the slower motions. The same ^2^H NMR measurements,^13^ that were used to estimate the residence time of water in the cellulose slit, also provide an estimate of 0.8 for the dynamic order parameter, which is close to the highest order parameters obtained here despite the limitations in drawing direct comparisons between the simulated ideal slit pore system and experimental fiber networks.

Reorientational (Figure 5) and translational (Figure 3) mobility decrease compared to their respective bulk values even at low confinement, and in both cases, the most confined systems exhibit orders of magnitude slower motions. However, reorientational mobility reaches its bulk value at a confinement where translation is still hindered. In addition, the type of confining surface (hydrophilic or hydrophobic) does not show any discernible difference in how it effects rotational mobility, which thus appears to be purely an effect of confinement.

As shown above, the slowing down of reorientational molecular motion sets in when *L* decreases down to around onenanometer. This is larger than typical interfibril distances in fibril aggregates, indicating that there will be water in plant cell walls and in cellulosic materials which is in a state of confinement-hindered mobility. Additionally, the order parameter suggests structural effects of confinement on top of the dynamical effects.

### Preferential Angles in the Orientation of Water Molecules

To better understand the nature of orientational order that is highlighted in one way by the order parameter *S*, we investigated the distribution of preferential orientations as an average across the trajectories. While *S* provides information on the temporal average, the distributions below reflect the statistics of the instantaneous configurations of confined water molecules.

Structured interfacial water with preferential orientations is often found in biological systems; where it plays a role in modulating biochemical interactions by screening dipole–dipole interactions as well as by shaping hydrogen-bonding networks.^26,61^

Here, the molecular water orientation relative to the nearest confining surface is represented by the two angles the OH-bonds form with (i) the surface normal (θ), and (ii) the cellulose chain direction (φ) (see Figure 1). This latter feature is absent for isotropic surfaces such as graphene or silica.^63^ These angles are selected as representation in order to relate to the anisotropic structure of the cellulose surface.

Distributions of cos θ and φ calculated for all water molecules within the slit at different extents of confinement are presented in Figure 6. Note that due to the conversion to spherical coordinates, cos θ and φ were chosen since their distributions are completely flat for isotropically distributed bond vectors (i.e., random orientation) whereas the distribution of θ would not be uniform.^65^ Thus, for a system with random orientation, ⟨cos θ⟩ = ⟨φ⟩ = 0.

**Figure 6:**
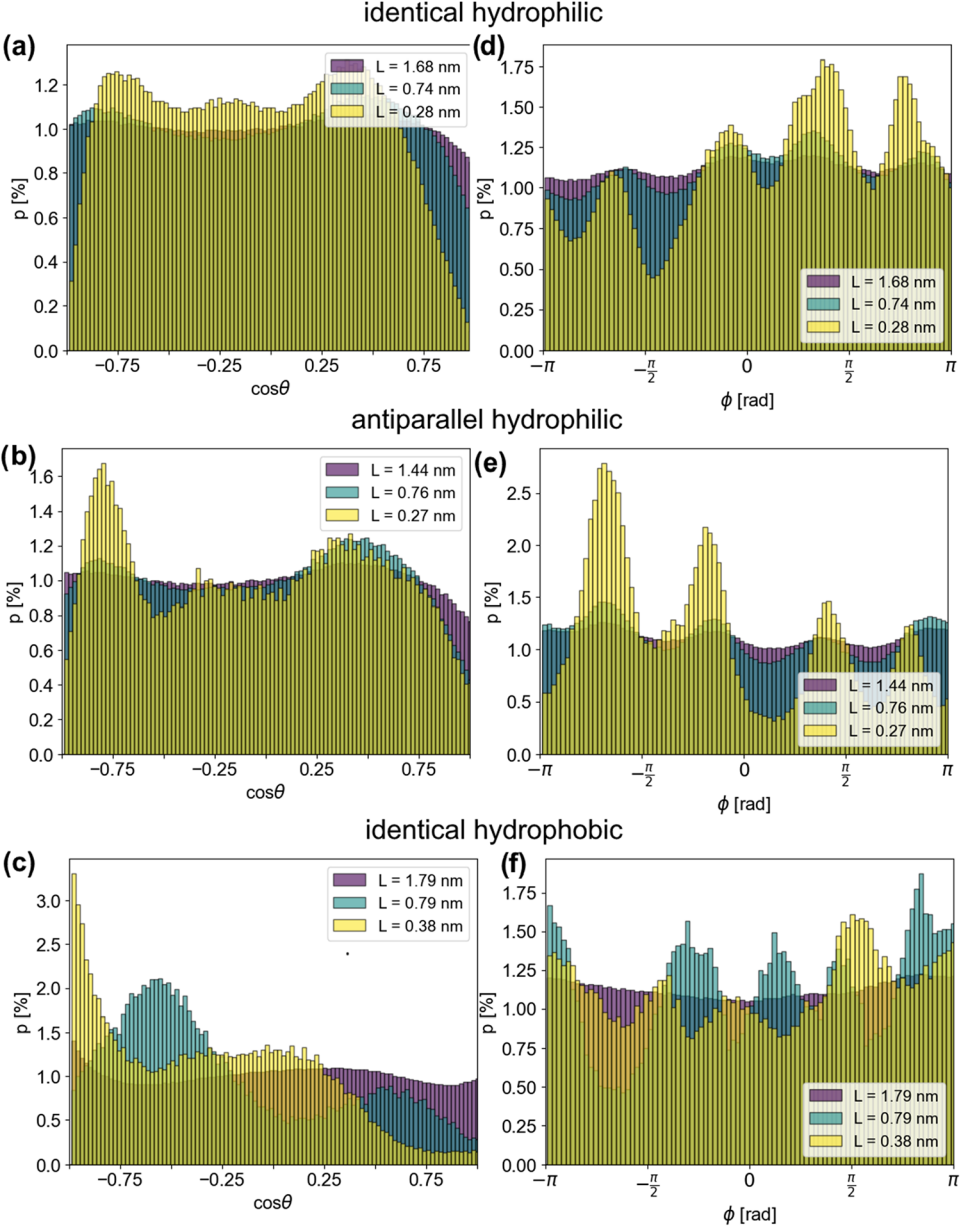
Distribution of cos θ (a–c) and φ (d–f) angles at selected extents of confinements, for identical hydrophilic (a, d), antiparallel hydrophilic (b, e) and identical hydrophobic (c, f) cellulose faces forming the confinement. Distributions are normalized to highlight the differences in the prominent peaks.

As the confinement in Figure 6a–c is increased, the multimodal cosθ distribution flattens out as the available space for water molecules increases. However, for the case of a hydrophilic confining cellulose surface, the distribution is skewed even at the lowest confinement. For this case, orientations where the OH bond vector points away from the surface are less common. As the orientation was calculated with respect to the nearest confining surface, the distribution is not expected to be symmetrical around cos θ = 0. In fact, our results regarding the angle with the surface normal are in agreement with the results of Watermann and Sebastiani^66^ and are a consequence of the known directional preference for cellulose-water hydrogen bonds formed at the interface.^67,68^ The distribution changes significantly for water confined by hydrophobic cellulose (Figure 6c), where there is a preference toward cos θ = −1 in highly confined systems,^69^ instead of two distinct preferential angles. Interestingly, when the size of the confined water slab changes there is a shift in the preferred angle. At high confinement (here, illustrated by data at *L* ≈ 0.28 nm), water molecules are in contact with both confining walls via secondary interactions, whereas as *L* increases (*L* ≥ 0.74 nm), interfacial water molecules are in contact with only one cellulose surface at a time, which is highlighted in the different preferential angles at angles above and below this threshold.

Figure 6d,e show that water has several distinct preferred orientations with respect to the chain direction when confinement is high (small *L*). Interestingly, it can also be seen that there is directionality in the sense that the distributions are skewed toward positive φ for parallel surfaces which reverses for the antiparallel case. This shows that the effect originates from the directionality of the cellulose chains. When the confining surfaces are hydrophobic (Figure 6c,f), preferential angles can still be seen in the distributions at high confinements, but there is no clear directionality. Upon increasing *L*, the peaks are slightly shifted, which is not the case for hydrophilic cellulose forming the confinement.

Unlike the preferential orientation, an indicator of structure, water dynamics is affected in a similar manner for all systems investigated and is thus an intrinsic feature of cellulose-water interactions. Flattening of the distributions (i.e., disappearance of preferential orientation) at *L* ≈ 0.8 nm corresponds well to the point where the reorientational correlation time τ and the order parameter *S* of the rotational reorientation become equal to those of bulk water, above *L* ≈ 1 nm. Therefore, water on the internal surfaces of a cellulose aggregate attains structure, as estimates of fibril–fibril separation distance generally fall under 1 nm.^49,50,56,57^

### Effect of Confinement on Water Density

The density provides additional information on the effect of confinement on the confined water layer. Figure 7 shows the average density as a function of *L*. At small *L*, there is not enough space for a complete monolayer of water and hence the density is lower than in the bulk. At around *L* = 0.3 nm, however, a monolayer with higher than bulk density is formed. This density is close to that of high-density amorphous ice and to the density of ice III, a tetragonal ice variant, both of which form under high pressure at around 77 K.^70^ For confinements above *L* = 0.7 nm, where there are more than 2 layers of water present, confinement appears to have no effect on the average water density, which becomes equal to that of the bulk liquid. This stands in contrast to the works by Giovambattista, Rossky, and Debenedetti,^28,29^ who studied water confined by model silica surfaces of different hydrophilicity. They found that already at a confinement of 1.6 nm, the average water density was significantly lower than in bulk. However, the effect was stronger for the more hydrophobic substrates and partially hydroxylated surfaces exhibited weaker effects. The greater extent of “hydrophobicity” is likely to cause the discrepancy between silica and cellulose. Molecular dynamics simulations of water contact angles on silica and cellulose substrates reveal that hydrophilic silica^71^ gives a similar contact angle as hydrophobic cellulose,^67^ around ∼30°. While the comparison is complicated by the fact that simulated contact angles depend strongly on the force field, both for the substrate and the water model, cellulose apparently behaves as a strongly hydrophilic substrate, regardless of which surface is considered. We conclude that below *L* = 1 nm of confinement structural properties are influenced. This is supported by the changes in the reorientational order parameter *S* (Figure 5). Average density data by themselves (Figure 7) are not able to pinpoint which way the water actually becomes structured although they can indicate the lack of a particular (dis)­order.

**Figure 7:**
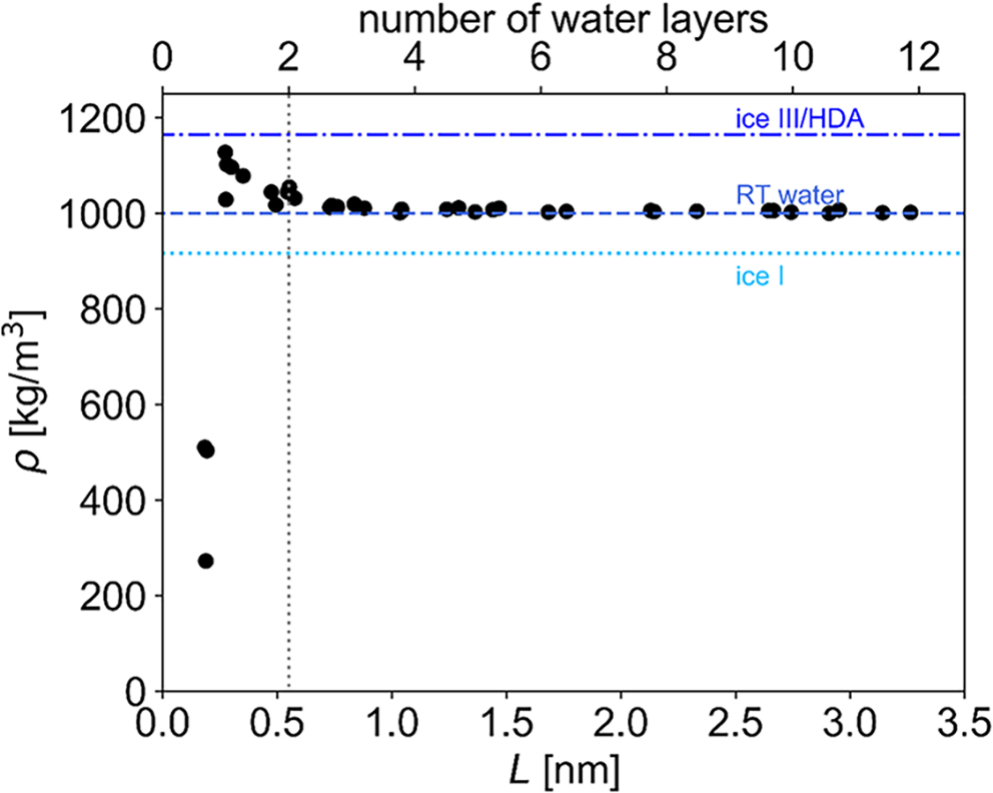
Density of the confined water as a function of confinement. RT water refers to room temperature water, HDA refers to high-density amorphous ice.^70^

On the local scale, the formation of structured hydration shells is a well-known phenomenon, which strongly affects interactions in hydrated systems. The formation of a water layer near the cellulose-water interface with a higher density than bulk was observed early on using molecular dynamics.^72^ The (1–10) crystalline face resulted in an interface with slightly higher density than the (110) face or the (100) hydrophobic face. Bulk density is reached at about half a nanometer from the cellulose surface in either case.

Figure S13 shows examples of the partial density profile at different *L*. With the exception of very high confinement where there is no complete water layer, there is no apparent change in the location, height, and width of the density peaks with confinement. This is a nontrivial observation, as water has been shown to form more pronounced density peaks upon increasing hydrophilic confinement, while there was no such effect in hydrophobic confinement by model fullerenes.^73^ Comparing mass density and number density profiles in the slit pores, one can observe that the two do not completely overlap; mass density has more prominent peaks (because mass density is more sensitive to the orientation of the water molecules). However, the average density trends are unaffected by which metric is used (see Figures S14 and S15).

At high confinement the confined water experiences driving forces toward structuring from both sides. Although this should *per se* not increase density, the preferential orientations detailed earlier suggest increased order in those systems. Effects on both local and average density appear to be more short-range than effects on mobility, since they are apparent only when less than two layers of water are present between the confining planes. Density effects further point toward the amphiphilic nature of cellulose; as average density behaves in a way that is observed in hydrophilic confinement, but on the other hand local density remaining unaffected by confinement is a property typical for hydrophobic confinement.

## Conclusions

A novel and rigorous definition of confinement in a water-filled slit bordered by cellulose surfaces has allowed us to investigate in detail the structural and dynamical effects of confinement on interfacial water down to very thin water layers. Both translational diffusion and rotational reorientation slow down as water becomes more confined, even more steeply so upon the water layer becoming thinner than bimolecular. Water also attains a preferential molecular orientation whose exact form depends on which cellulose surface (the more hydrophilic (110) and (1–10) or the more hydrophobic (100) crystalline face) is in contact with the water. Both the slowing down of molecular motions and the orientational order inferred/derived from our simulations are in good agreement with relevant ^2^H NMR parameters obtained earlier in microcrystalline cellulose.^13^

In this regime, where the dynamics slows down, the water density shows a nonmonotonic dependence on the confinement. Intriguingly, the highest observed density is comparable to that of ice formed under high-pressure (such as ice III), which is seldom observed in ambient conditions. It has been shown previously that water confined to the internal surfaces of a cellulose fibril aggregate has lower free energy than water surrounding the aggregate, therefore its presence is thermodynamically favorable.^20^ At first glance this feature is a bit contradictory since the preferential orientation of water molecules leads to a massive loss of configurational entropy. The significant increase in average density for a monomolecular layer would make this apparent contradiction more pronounced. Strong secondary interactions between water and cellulose may provide an explanation, as we speculate that favorable enthalpy in the confined system could overcome the entropic penalty. This hypothesis should be looked into it in more detail in the future.

Persistent orientational order even in water layers as thick as *L* ∼ 0.8 nm suggests that secondary interactions between the cellulose surfaces might not be completely screened, further reinforcing the notion of secondary interactions being important. A connection can be drawn to the way interfacial water has been shown to impact the deformation mechanism of hydrated cellulose in atomistic simulations, where the lack of screening at high confinement explains why the appearance of the first layer of water between cellulose crystals does not lead to a major compromise in shear modulus and strength.^9^ Understanding the behavior of water at the interfibril interfaces can help us rationalize how moisture impacts mechanical properties and deformation mechanisms.^23^ Cellulose appears to be rather unique in this respect, as our results shows that water properties are more strongly affected than in, e.g., silica or graphene. An open question is how much of our observations can be attributed to confinement versus specific cellulose-water interactions, and whether these two effects can be distinguished in biologically or technically relevant systems.

## Supplementary Material



## Notes

Comparison of different definitions for the extent of confinement; example fits to the MSD and RACF curves; covariance analysis; details on the stretched exponential and density profile calculations (DOCX) (PDF)

## Abbreviations

MD: molecular dynamics
MSD: mean square displacement
NMR: nuclear magnetic resonance spectroscopy
RACF: rotational autocorrelation function
