# Supplementary material for: Icing in the Cake: Water in Nanoscopic Confinement by Cellulose
Source: J Phys Chem B. 2025 Nov 19;129(47):12348–57. doi: 10.1021/acs.jpcb.5c06900 (PMC12670412; doi:10.1021/acs.jpcb.5c06900)
Supplement: Supplementary file 1 [file jp5c06900_si_001.pdf]

Supporting information for *Icing in the Cake: Water in Nanoscopic Confinement by Cellulose*

Alíz Lelik[a,c], Lars Berglund[a,c], István Furó[b,c], Jakob Wohlert[a,c]*

[a]: Department of Fiber and Polymer Technology, KTH Royal Institute of Technology, SE-10044 Stockholm, Sweden

[b]: Department of Chemistry, KTH Royal Institute of Technology, SE-10044 Stockholm, Sweden

[c]: Wallenberg Wood Science Center, KTH Royal Institute of Technology, SE-10044 Stockholm, Sweden

Comparison between diffusion in the x and y directions

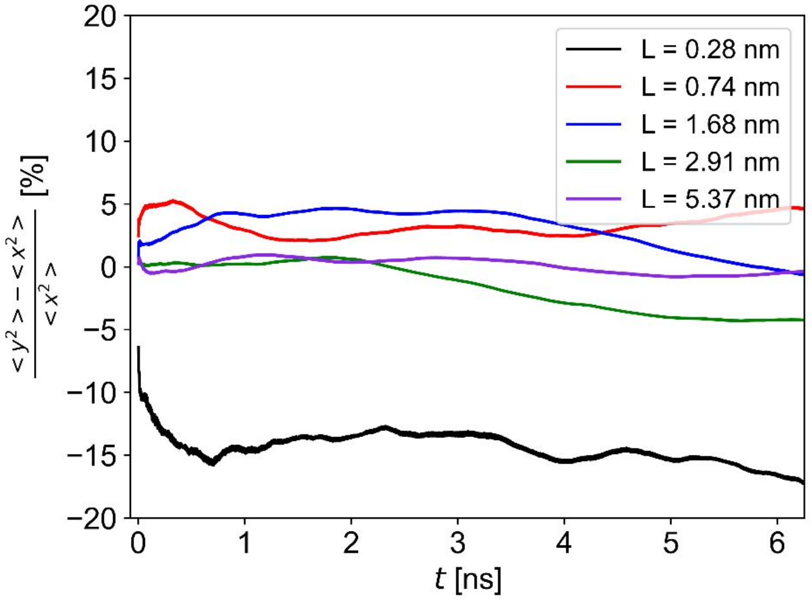

Figure S1: Difference between the mean square displacements in the x and y directions, as a function of time, at selected extents of confinement

Comparison of different definitions for the extent of confinement

There exist several definitions of the extent of confinement between studies of confined water in systems with slit-pore geometries.[1–3] The most common way to define it is based on geometry, as the distance between the atoms making up the confining surfaces. This definition is not well applicable to surfaces with atomistic scale roughness, and does not consider that there may be areas near the surface which are not accessible to the water molecules, due to sterical exclusions.

An other approach, adopted by Giovambattista et al.[2,4] is to define an effective plate distance based on the probability distribution of the water molecules between the two plates - although it is noted, that this method does lead to an underestimation of the volume that is actually available. This method, and other methods based on the density profiles take into consideration the known effect the hydrophobicity of the confining surfaces has on the accessible area for the water molecules.[5]

The extent of confinement (L) in this study was defined approaching the question based on dynamics, also with the aim of considering accessibility of the space for water. The distance between confining walls can be left as a parameter to be fitted in eq 3 (in the main text), while fitting to the mean square displacement data in the direction perpendicular to the cellulose surfaces. Therefore, this parameter can also be used to describe the volume available for the water to diffuse into, as a definition of the extent of confinement. While fitting to this expression with an infinite sum, instead of considering a set number of terms; terms were considered as long as their absolute value added to the value of $<x^2>$ is larger than 10^{-12} m^2.

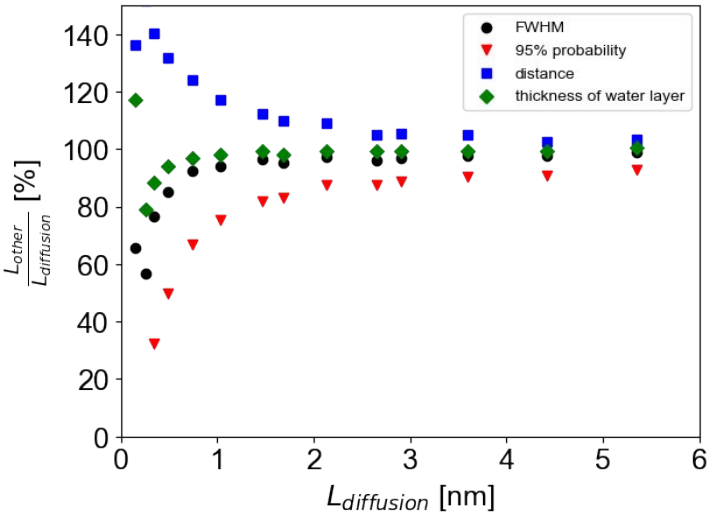

Figure S2 Comparison between the different definitions of the extent of confinement normalised to the diffusion-based approach

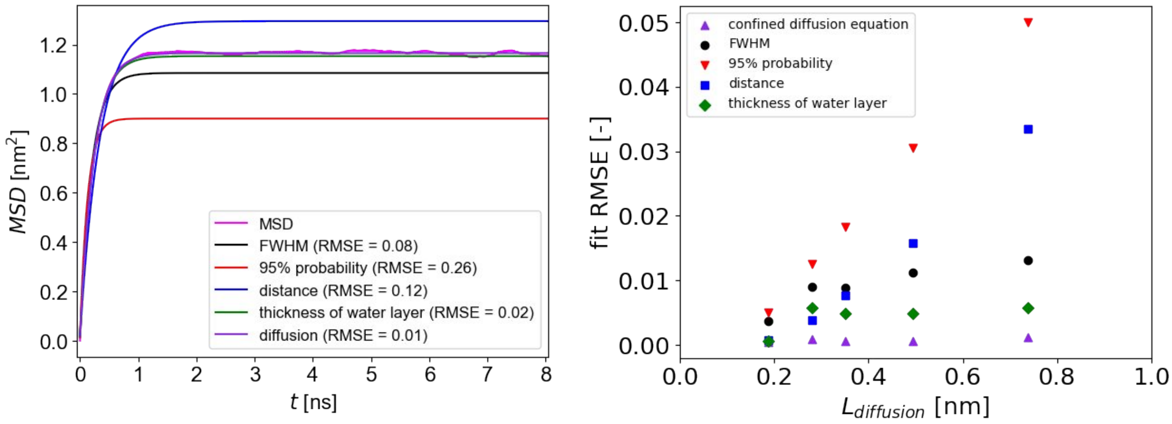

a) b)

Figure S3 Comparison between the results of fitting to the diffusion equation using different approaches of defining L for an example system (a) and the root mean square error of these fits in different systems, where $L_{diffusion}$ is used as identification (b)

In practice, the difference between L's obtained from different definitions can be rather large, therefore choosing the most appropriate definition for studying confinement effects is critical. To compare the different approaches, four different ones based on geometry or the density

profile of water molecules to define the extent of confinement are compared with the diffusion-based approach, where $L_{diffusion}$ is left as a fitting parameter. These approaches are:

- Using the full width at half maximum (*FWHM*) of the density curve as $L_{density}$
- Calculating the width of the space where the water molecules are found with a 95% probability based on integrating the partial density profile
- Taking the average distance in the coordinate normal to the surface between the C6 atoms in the surface cellulose chains
- From the known number of water molecules, the cross-sectional area of the simulation box, and the number density of water molecules in the system, calculating the theoretical thickness of the water layer. Throughout these calculations, an assumption has been made that the density of water in the system is a uniform 1000 kg/m^3

Using $L_{diffusion}$ as a baseline for 100%, we can observe that there are substantial differences in the extents of confinements obtained for the same systems, especially at small confinements (figure S1).

To assess which definition is the most suitable for the purposes of interpreting the results of this study, the quality of the fit between the calculated MSDs and the fitted datasets using the different definitions was compared using the root mean square error (RMSE) value (figure S3b). Figure S3a illustrates the problem that arises when using other L's – the large systematic error in the plateau of the MSD curve. Naturally, the fit using two parameters is expected to be better than any of the fits containing only one parameter, but this example comparing the quality of the fits shows that when talking about the dynamics, definitions based on structural parameters give less satisfying results than approaching the area accessible to water molecules based on their dynamic characteristics, especially in the case of highly confined water. The approach based on the theoretical density of the water has the second lowest RMSE value, however based on the results detailed in the main body of the text, we can see that the assumption of uniform bulk density is far from true. Thus, for the purposes of interpreting the results of this study, the diffusion-based definition was chosen. It must be noted, that the definition of "distance from the cellulose surface" brings along similar problems as defining the extent of confinement. In this study, the distance from the surface (z) was chosen in a way, that the at the center of the slit-pore, $z = L_{diffusion}/2$.

Example fits to the MSD and RACF curves, covariance

Below, a set of MSD and RACF curves are presented, with their respective fitted expression plotted alongside them, to showcase the quality of the fit at different extents of confinement. The extents of confinements to display were chosen to show representative examples. The covariance of the parameters D and L, and the parameters τ and β was evaluated based on a Monte Carlo method of adding random noise to the dataset and repeating the fitting 100 times, the resulting set of parameters are also shown here.

The shape of the spread around the original data pair shows, that D and L are highly independent of each other at all extents of confinement.

L = 0.28 nm

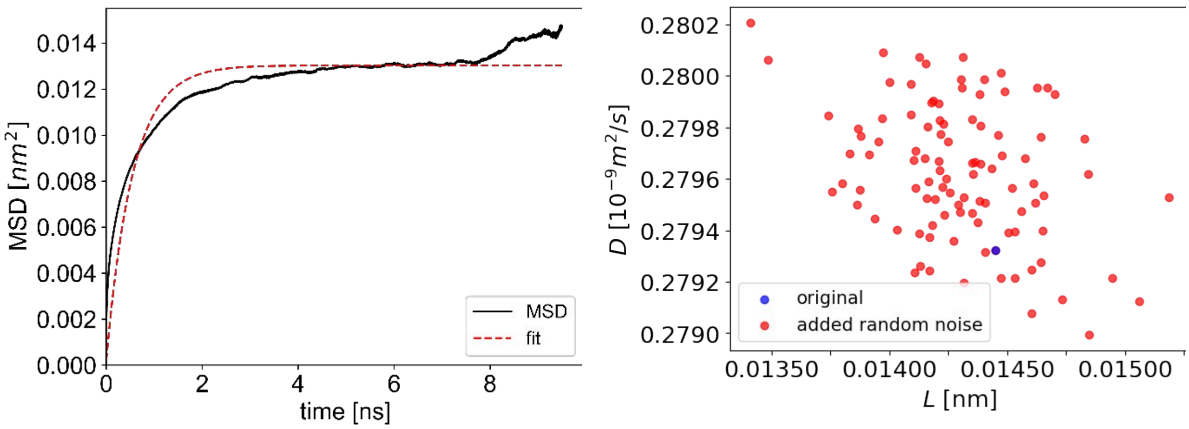

Figure S4

L = 0.49 nm

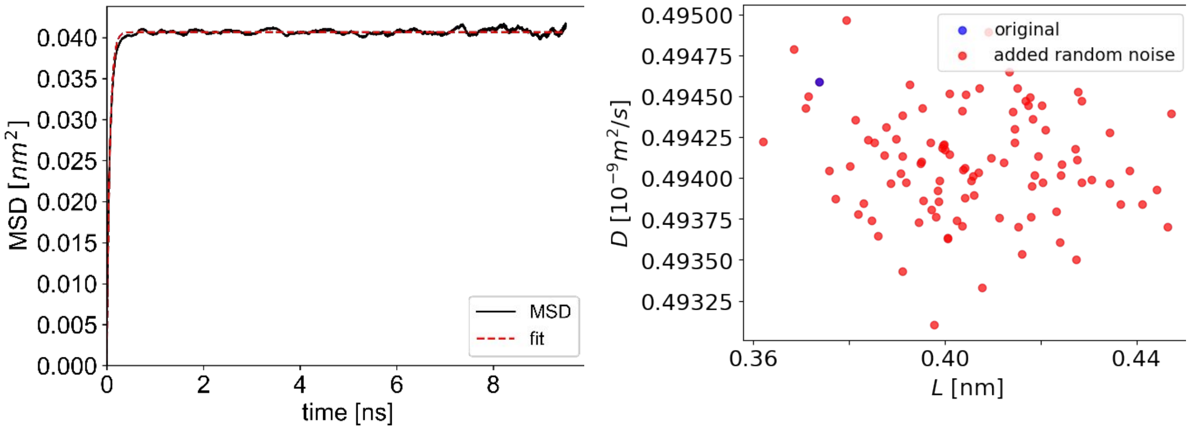

Figure S5

L = 1.03 nm

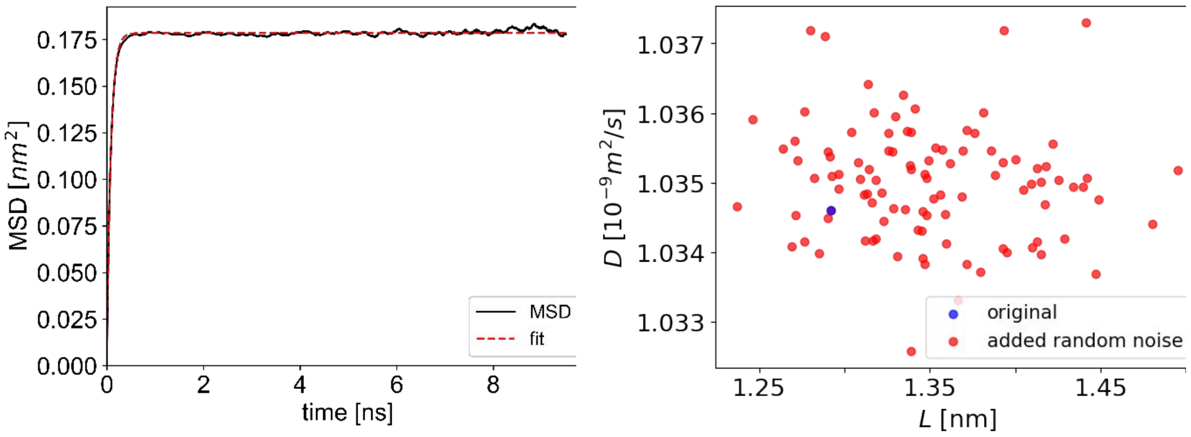

Figure S6

L = 1.68 nm

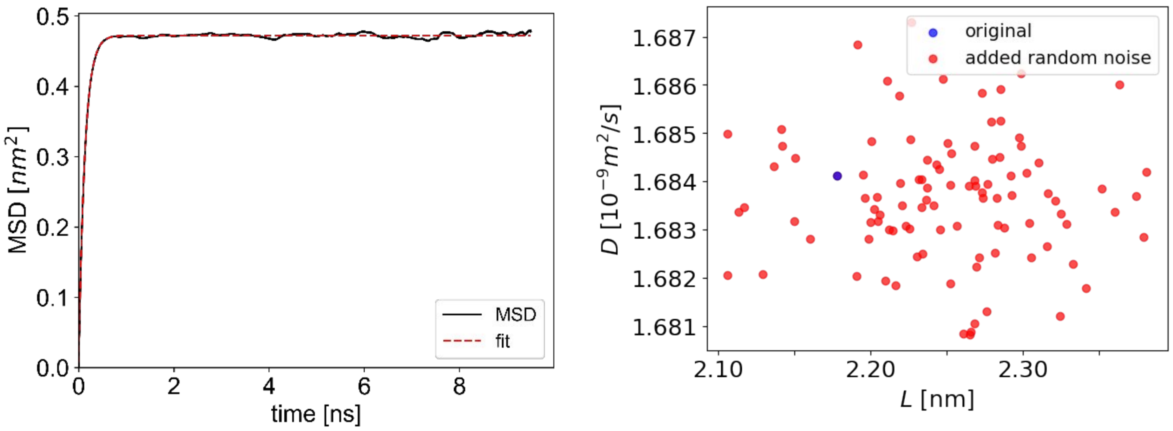

Figure S7

Unlike the parameters of the diffusion equation, τ and β are independent at low extents of confinement, but have a strong correlation at high L values. These results are expected for a stretched exponential fit, where the parameters are not a direct representation of a physical property, rather model parameters for comparisons.

L = 0.28 nm

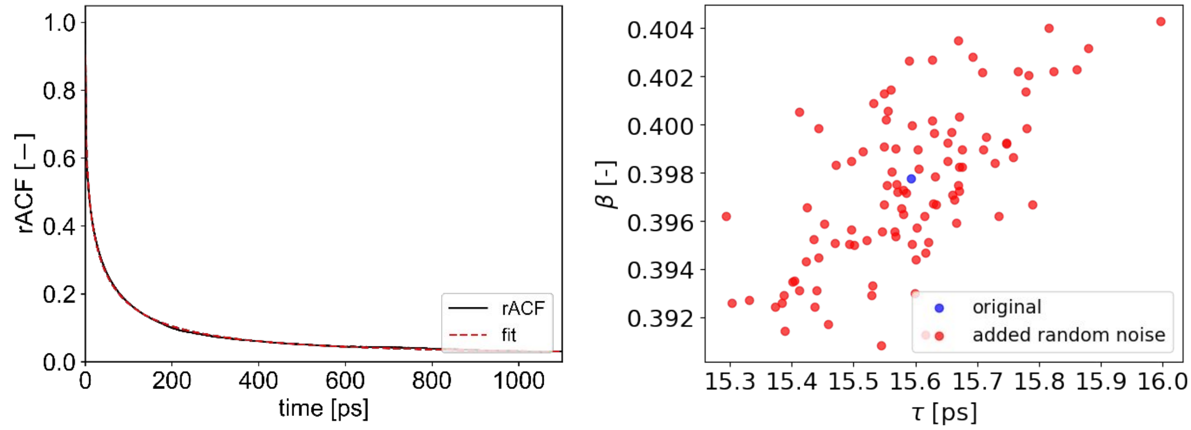

Figure S8

L = 0.49 nm

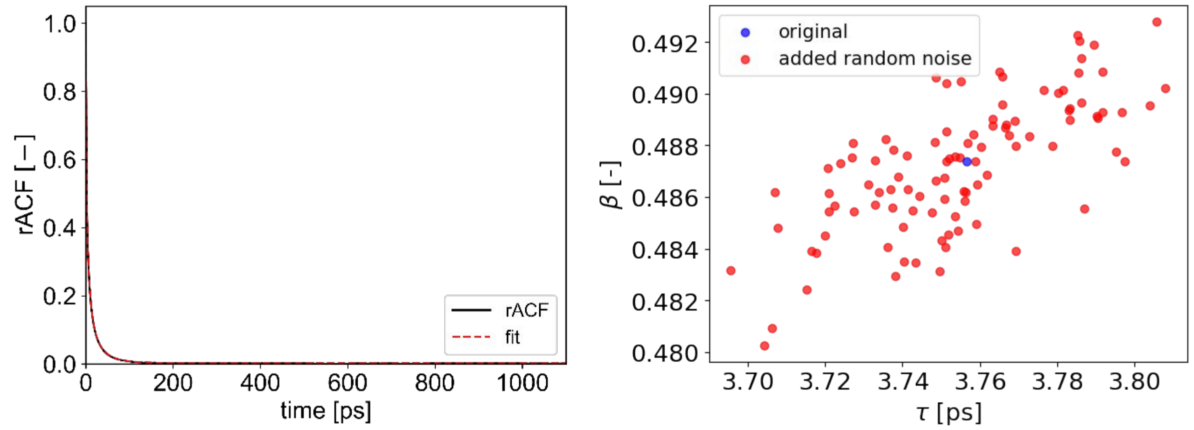

Figure S9

L = 1.03 nm

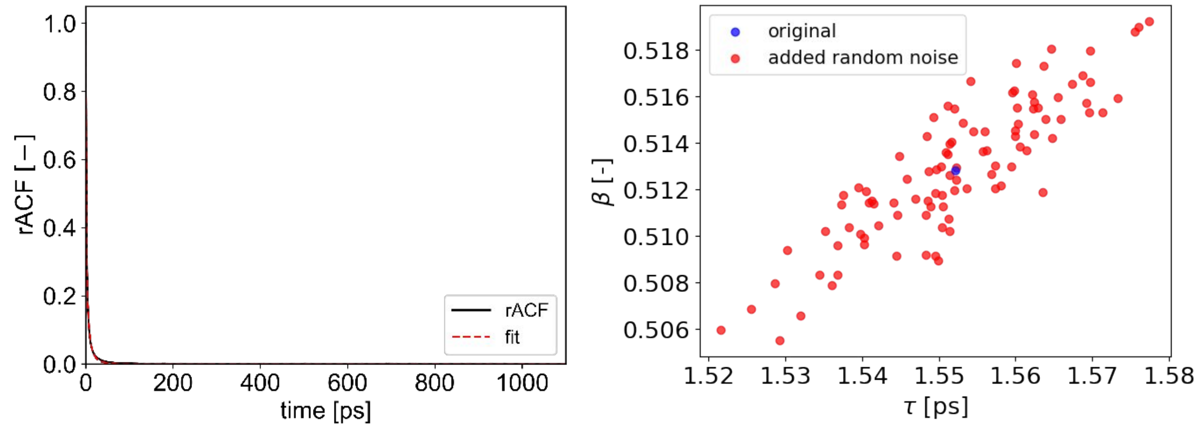

Figure S10

L = 1.68 nm

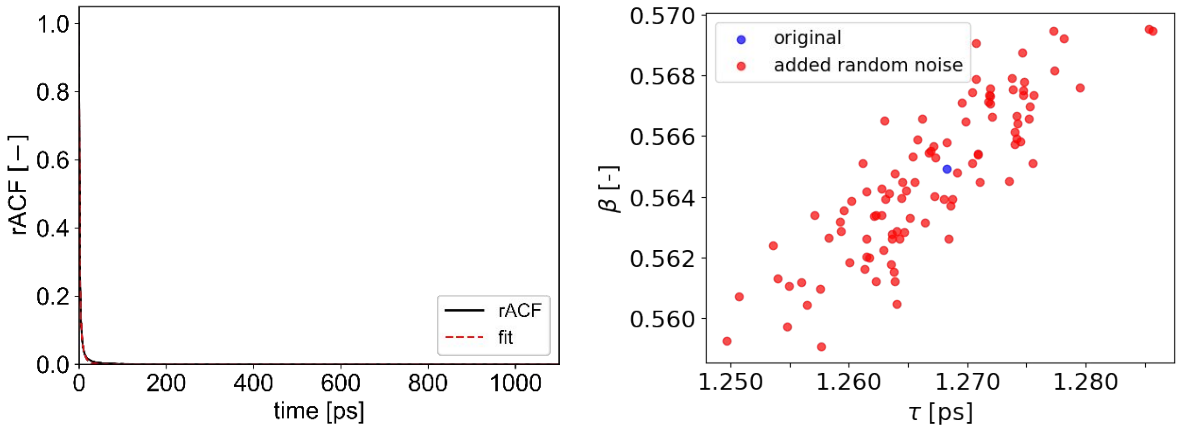

Figure S11

Stretched exponential

A stretched exponential function can be fit well to *C(t)* if the plot of log*t* against log(-log(*A(t)*) is linear, where $A(t) = (C(t)-S^2)/(1-S^2)$. This plot is shown in FigureS12 for selected extents of confinements.

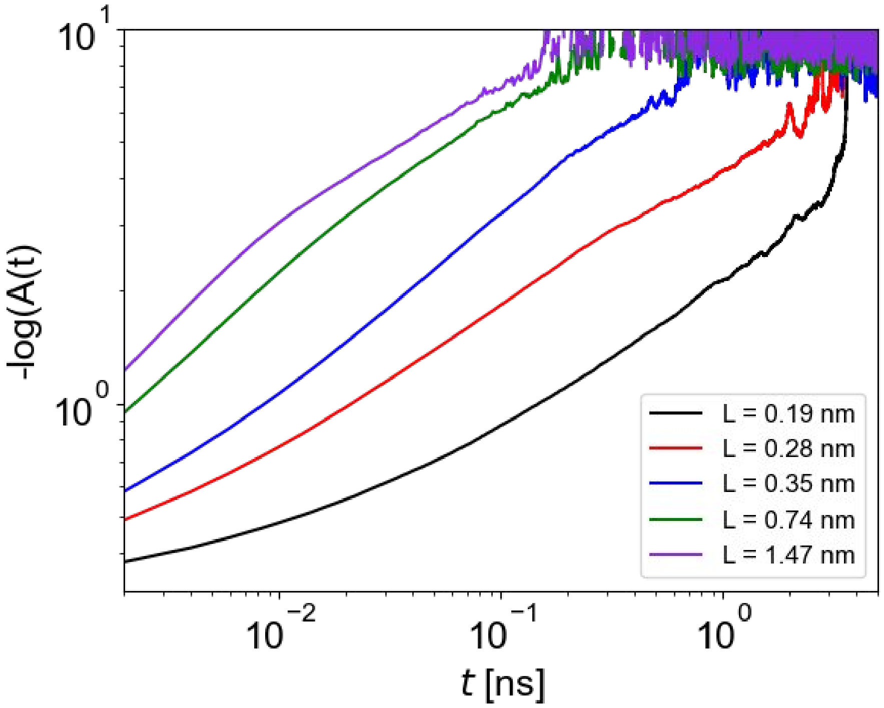

Figure S12: log(-log(A(t)) plot

One can observe here that the linear approximation is somewhat fitting in the regime of *t*=0.01 – 1 ns, where the decay is the most significant, before it plateaus at a constant value, except at the lowest extent of separation between confining surfaces, where the motions are expected to become irregular.

Partial density profiles, comparison of mass density and number density

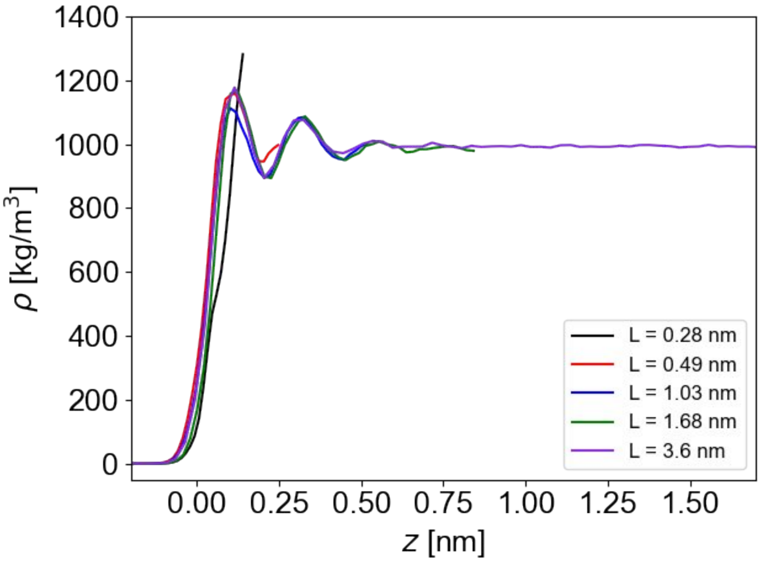

Figure S13: Partial density profile of the confined water at different extents of confinement

The density profiles in these systems can be calculated from the simulations both as the mass density and the number density of the water molecules. The mass density is somewhat sensitive to the orientation of the water molecules, meaning that if there is a preferential orientation in the θ angle, the calculated mass and number density profiles may not completely overlap. Instead, the mass density will be higher where the O atoms point, which in this case is close to the surface.

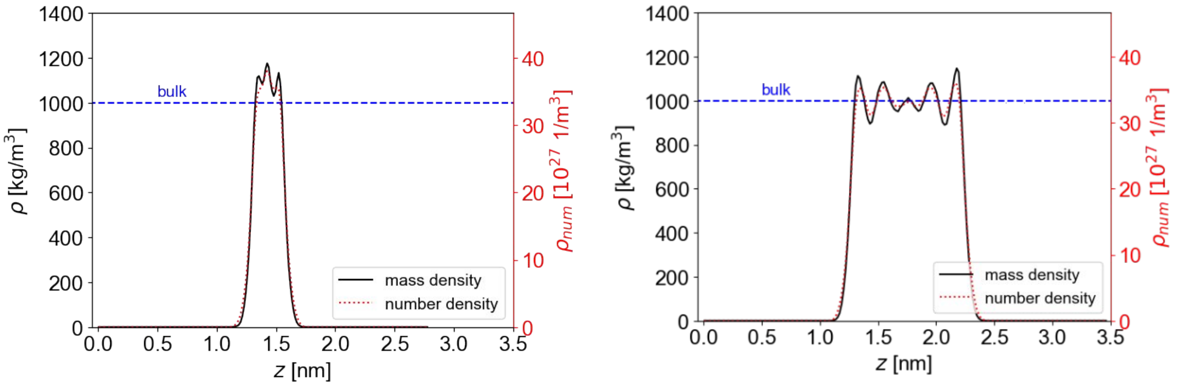

Figure S14 Comparisons of the density profile calculated from mass- and number density

The calculation of the average density is only slightly affected under L = 1 nm, but the overall trends remain the same.

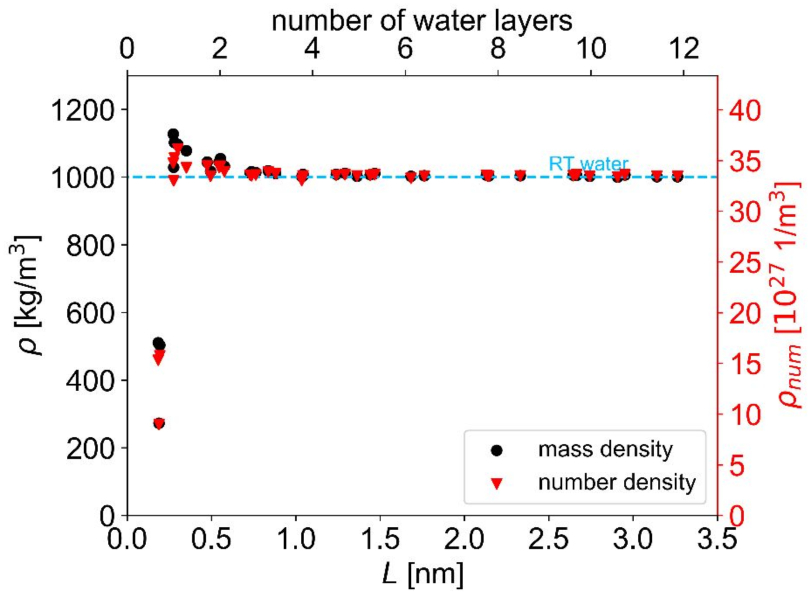

Figure S15: Average number and mass density calculated across the pore

References

(1) Giovambattista, N.; Rossky, P. J.; Debenedetti, P. G. Effect of Temperature on the Structure and Phase Behavior of Water Confined by Hydrophobic, Hydrophilic, and Heterogeneous Surfaces. *J. Phys. Chem. B* **2009**, *113* (42), 13723–13734. https://doi.org/10.1021/jp9018266.

(2) Giovambattista, N.; Rossky, P. J.; Debenedetti, P. G. Effect of Pressure on the Phase Behavior and Structure of Water Confined between Nanoscale Hydrophobic and Hydrophilic Plates. *Phys. Rev. E* **2006**, *73* (4), 041604. https://doi.org/10.1103/PhysRevE.73.041604.

(3) Zangi, R.; Mark, A. E. Bilayer Ice and Alternate Liquid Phases of Confined Water. *J Chem Phys* **2003**, *119* (3), 1694–1700. https://doi.org/10.1063/1.1580101.

(4) Wang, C.-W.; Kuo, Y.-W.; Zeng, J.-R.; Tang, P.-H.; Wu, T.-M. Confinement Effects on Reorientation Dynamics of Water Confined within Graphite Nanoslits. *J. Phys. Chem. B* **2024**, *128* (39), 9525–9535. https://doi.org/10.1021/acs.jpcb.4c03898.

(5) Brovchenko, I.; Geiger, A.; Oleinikova, A.; Paschek, D. Phase Coexistence and Dynamic Properties of Water in Nanopores. *Eur. Phys. J. E* **2003**, *12* (1), 69–76. https://doi.org/10.1140/epje/i2003-10028-4.
